# Learning from the parallel pathways of Makers to broaden pathways to engineering

**DOI:** 10.1186/s40594-017-0098-8

**Published:** 2018-03-02

**Authors:** Christina Foster, Aubrey Wigner, Micah Lande, Shawn S. Jordan

**Affiliations:** 1 Arizona State University—Polytechnic Campus, Mesa, AZ USA; 2 Michigan State University, East Lansing, MI USA

**Keywords:** Engineering education, Making, Engineering, Pathways

## Abstract

**Background:**

Makers are a growing community of STEM-minded people who bridge technical and non-technical backgrounds to imagine, build and fabricate engineering systems. Some have engineering training, some do not. This paper presents a study to explore the educational pathways of adult Makers and how they intersect with engineering. This research is guided by the following research questions: (1) What can we learn about the educational pathways of adult Makers through the lens of constructivist grounded theory? and (2) How do the educational pathways of Makers intersect with engineering? This study relied on qualitative interviews, using artifact elicitation interviews and constructivist critical incident technique interviews, of 42 adult Makers.

**Results:**

Through inductive analysis of a collection of interviews with Makers, a theme emerged where Makers from different educational backgrounds and with different careers (e.g., art, STEM, business) were making artifacts that had similar purposes. We present two cases of parallel pathways, (1) musical artifacts and (2) large-scale interactive artifacts, to demonstrate the multiple, parallel life pathways that Makers take to making their artifacts and the contextual events and activities that are critical to the direction of these pathways.

**Conclusions:**

The stories and life pathways of adult learners engaged in Making can offer valuable insight into how we might identify practices that promote the access and success of a larger and more diverse population of students for engineering. Makers are engaged in activities that embody the Engineer of 2020 (e.g., lifelong learning, creativity, and practical ingenuity). By studying Makers, we can consider the multiplicity of pathways into engineering majors and careers.

## Introduction

Engineering is increasingly understood as a lifelong learning pathway rather than an event that happens only in a university setting. The National Academies *Engineer of 2020* (National Academy of Engineering 2004) and ABET, the US accreditation body for engineering programs (ABET 2012, 2014, 2015) have both identified a wide range of qualities that are vital for future engineers. The identified qualities include the ability to engage in lifelong learning, function creatively, work across disciplines, practical ingenuity, and the ability to communicate with broader audiences in addition to maintaining technical expertise in engineering fields. These qualities are important for, and learned within, a wide range of practices. To better understand how engineering education manifests during a lifetime, and how engineering skills and mindsets can be acquired later post-college, we seek to understand how skills, knowledge, and tacit knowledge are built. This paper explores one method for discovering how engineers are made, both through traditional and non-traditional processes.

To see what events and skills make an engineer, we explored the life pathways of Makers, a self-identified group of creatives that bridge across many areas of technical and non-technical expertise. A combination of qualitative techniques was used for data collection including a screening questionnaire, artifact elicitation interviews, and critical incident interviews. This paper expands on the collection methods used in order to inform others of possible approaches for understanding the skills learned and pathways taken by a sector of the adult community who embodies many of the qualities important to the engineer of the future.

## Background

### What is a Maker?

A Maker is a modern-day tinkerer and hands-on doer and fashioner of stuff. Makers are do-it-yourself-minded individuals participating in informal communities (doing it with others) that support and celebrate building and prototyping technical proof-of-concept exploration and ad hoc product development. The label “Maker” is a self-determined one assigned by affinity with or involvement in a larger Maker community. The range of expertise could be large, but novices and experts alike share an enthusiasm and appreciation for building and creation. Individuals and groups embark on projects of all sorts, led primarily by their interests and curiosities, informed by their skills or the skills they want to learn. Making comes from an imaginative, creative mind-space, and is often done outside the confines of established engineering education curricular activities. For example, one might make creative efforts like fire-breathing robots as performance art, combining contributions from community members with electrical, mechanical, and embedded systems know-how. Makers are rich in creative confidence, with their expertise sometimes lying in the ability to learn new skills as needed rather than already possessing immediate solutions to the problems that they encounter.

The Maker Movement is an emerging and developing sub-culture that values the tinkering, hacking, re-making, and creating of artifacts that require the use of both technical and creative skills. Making has a do-it-yourself ethos and is historically rooted in efforts like *Popular Mechanics* magazine which demystified everyday stuff for hobbyists and the *Whole Earth Catalog: Access to Tools* (1972) which surveyed everyday tools for the counterculture movement of the 1960s. Additional real-world touchstones are the growth of Radio Shack stores, and the 1980s television program *MacGyver* where the lead character would resolve each episode’s predicament by fashioning an escape plan out of found objects (Zlotoff 1985).

Technology and sharing of information via the Internet have greatly increased the ability for smaller communities with shared interests to coalesce and grow. Makers participate in communities of practice (Sheridan et al. 2014, Wegner 2010), gathering with like-minded individuals and groups to learn skills and share interests and affinities. They populate maker spaces and hacker spaces (Tweney 2009) to gather with other Makers. A significant part of such participation is to benefit from opportunities to continually learn from, teach and mentor other Makers. Makers exemplify the collaborative model of *additive innovation* by seeking and offering inspiration in their community, sharing and learning recipes with others, iterating on their own designs, and sharing artifacts of their designs back with the community to inspire others (Jordan and Lande 2015).

### What is an engineer?

The work of an engineer is framed by the nature of engineering, the types of problems engineers solve, the knowledge and methods used to solve those problems, and the best practices that engineers should follow.

The nature of engineering stems from its name, as it was derived from the Latin root *ingenerare* meaning *to create*. Koen (1985) wrote: “To identify a situation calling for an engineer, seek a situation calling for change”. The changes engineers make have a distinct function; they are intended to create an optimum solution for a human or societal need (Koen 2003). Engineering’s purpose to create solutions is often conceptualized as problem solving (Sheppard et al. 2009). The types of problems that engineers deal with are *design problems* and are characteristically practical, achievable, ill-structured, context-specific, and have no definitive solution (Cross 2008; McKenna et al. 2011). Like engineering problems, their outcomes—engineering solutions—are not rigid. An engineering solution is the “best change within available resources” (Koen 2003) and can take many forms, including systems, processes, and artifacts.

The method that engineers use to solve problems is often referred to as engineering design and relies upon the use of heuristics (Koen 2003). Engineering design indeed is the common denominator across all engineered products and is what can be used to identify the presence of engineering activity (Koen 1985). Like the diversity among engineered artifacts, the engineering design process is complexly varied in the ways it is performed. As Bucciarelli (1994) pointed out, the engineering design process does not take one singular form. Pahl et al. (2007) also show that the engineering design process is a complex landscape of approaches, stages, principles, purposes, and traditions. Design is not stagnate, absolute, guaranteed, or deterministic. Rather, it is a dynamic, complex process that is a part of a greater feedback loop used by the engineer to know engineering and construct new engineering knowledge. However, across any variation of the engineering design process, the characterization remains the same. Engineering design is systematic, iterative, purposeful, creative, and social (ABET 2014; Bucciarelli 2003; Cross 2008).

To implement engineering design, engineers must converge various sources of knowledge. The knowledge needed by engineers has been categorized in different ways. For example, Figueiredo (2008) conceptualizes engineering knowledge as a 4D transdisciplinary model, with the four dimensions being basic sciences knowledge, design knowledge, social science knowledge, and knowledge from doing. In this model, the engineer must converge and internalize the knowledge found within these four dimensions in order to carry out the engineering method. Regardless of the model, engineers use knowledge to make decisions and build consensus during the engineering design process. Engineering knowledge enables engineers to reason through heuristics, with heuristic reasoning as “not regarded as final and strict but as provisional and plausible only, whose purpose is to discover the solution to the present problem” (Polya, 2014). This enables engineers with the flexibility needed for determining the most logically compelling argument for the context. Acceptance of a heuristic depends on whether it works and is useful in a specific context. As engineers create and use heuristics to guide their work, they gain expertise that informs their work. This contributes to the consensus-building nature of engineering which is used to determine best practices (Koen 1985). Determining what is state of the art through heuristic reasoning guides engineering practice to its goal—to develop an optimum solution for the design problem at hand.

The best practices of engineers are reflected by codes of conduct. These codes “acknowledge the overall mission of the profession as contributing to human welfare” and that engineers must be competent (Sheppard et al. 2009). The attributes that engineers must possess to do their work are practical ingenuity, strong analytical skills, discovery and design, creativity, communication, accountability, mastery of principles of business and management, professionalism, and life-long learning (ABET 2014; NAE 2005).

### Broadening engineering pathways

A more inclusive vision of engineering crossed with Making could build future engineering capacity by engaging individuals not attracted to the traditional engineering community. Additionally, the actions of Makers as citizen engineers could raise awareness among the public about engineering. The Center for the Advancement of Engineering Education’s Academic Pathways Study (Atman et al. 2010) studied undergraduate persistence in engineering and found two groups of students with different motivations for engagement. The first group sought financial security, aiming for graduation while overcoming barriers of foundational math and science courses. The second group approached their studies with intrinsic psychological motivation, seeking meaning and impact through their studies. Students in the second group represented myriad races, ethnicities, and socio-economic statuses (Atman et al. 2010).

The purpose of our research is to change the conversation to highlight the possibilities for the second group, which includes underrepresented minorities. We acknowledge the differences between engineering students, practicing engineers, and Makers but find the possible overlaps and stories of pathways below to have the potential to inform transformational change in our field. There are considerable benefits to STEM education, as well as resulting societal benefits, for those who have influence over student decisions to have an appreciation of the multiplicity of pathways into STEM careers. Likewise, pathways can show the value of technical literacy based on Making activities. This is especially true for underrepresented groups to make the case that they are evident in the population of people already doing engineering and other STEM activities.

## Methods

This paper presents a study to explore the educational pathways of adult Makers and how they intersect with engineering. This research is guided by the following research questions and research design:

1. What can we learn about the pathways of adult Makers through the lens of constructivist grounded theory?
2. How do the educational pathways of Makers intersect with engineering?

This research, guided by RQ1 and RQ2, is situated within a broader 4-year study exploring Making as a means of engaging current engineering students as well as adults and pre-college students in the engineering landscape described in *The Engineer of 2020*, *ABET a-k*, and *21st Century Skills*.

**Fig. 1:**
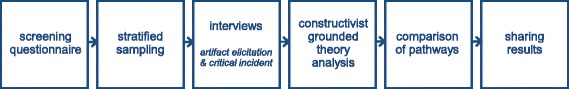
Research process

There is currently little known (Corbin and Strauss 2014) about what Makers know and their pathways; therefore, the methodological framework must guide the study to be grounded in Makers’ voices and perspectives. The design of the study was formed with Crotty’s (1998) four elements of a research study (epistemology, theoretical perspective, methodology, and methods), which laid the groundwork for the methods implemented in this study and guided the actions and perspectives of the researchers. Specifically, the study relied upon the methodology of constructivist grounded theory (Charmaz 2006), whereby the researcher is the author of participant’s voice and meaning. The methodological framework was informed by the epistemological perspective of constructivism (Piaget 1996) to emphasize that knowledge is constructed through human-world interaction and, specifically, to guide the research study toward understanding how and what Makers learn through their actions to create. Additionally, the methodological framework was informed by a theoretical perspective of constructionism (Martinez and Stager 2013; Papert and Harel 1991), whereby meaning is created through constructing and sharing artifacts. This theoretical perspective focused the study on how Makers create meaning through the design and sharing of their creations. The design of the research study guided the implementation of the methods. The methods specifically relied upon screening questionnaires, artifact elicitation interviews, and critical incident technique interviews in order to understand Makers’ creations, knowledge, and skills learned by creating and to study Makers’ attitudes about and pathways intersecting with engineering. The research design, organized through Crotty’s (1998) framework, is summarized in Table 1, and the overall process is summarized in Fig. 1.

**Table 1:** Elements of a research study by Crotty (1998)

	Definition	Selected	Rationale
Epistemology	Theory of knowledge	Constructivism knowledge is constructed through human-world interaction (Piaget 1996)	To understand how and what Makers learn through their creations
Theoretical perspective	Philosophy that informs methodology	Constructionism meaning is created through constructing and sharing artifacts (Martinez and Stager 2013, Papert and Harel 1991)	To understand how Makers create meaning through the design and sharing of their creations
Methodology	Design connecting methods to outcomes	Constructivist grounded theory researcher is the author of participant’s voice and meaning (Charmaz 2006)	Little is known (Corbin and Strauss 2014) about what Makers know and their pathways. Methods must be sensitive to study objectives: to understand what Makers learn and how their pathways intersect with engineering.
Methods	Implementation of methodology	Screening questionnaire Artifact elicitation interviews Critical incident technique interviews	To screen potential participants To understand Makers’ creations/knowledge/skills learned by creating To study Makers’ attitudes about and pathways intersecting with engineering

Following the research design, researchers began with administering screening questionnaires to conduct stratified sampling. For those potential participants that were selected for the study, interviews were conducted and analyzed according to the methodological framework. Through inductive analysis under the constructivist grounded theory, the pathways were compared and results were shared. Figure 1 summarizes this process.

### Population and sampling

This study relied upon a population of adult Makers in order to understand how their pathways intersect with formal engineering education. We sought out Makers who participate in Maker Faires. A total of 42 Makers who exhibited at two flagship Maker Faires participated (see Table 2).

**Table 2:** Participant recruitment (*N* = 42) and interviews (participant pool, location, date)

	Round 1	Round 2	Round 3	Round 4
Makers	7	9	12	14
Location	New York	Bay Area	New York	Bay Area
Date	September 2012	May 2013	September 2013	May 2014

The Bay Area Maker Faire draws over 500 exhibitors annually. A stratified purposeful sampling strategy (Patton 2002) was used for initial selection of participants. Participants were selected to maximize variation across the strata described in Table 3 while oversampling for underrepresented groups and ensuring that all participants self-identify as Makers. This sampling strategy was appropriate to target the Maker population relevant to the research questions, whereas representative sampling may not provide a complete picture of knowledge, skills, attitudes, and pathways of Makers necessary to inform theory generation. Each participant received a monetary incentive for his or her time.

**Table 3:** Stratifications for purposeful sampling

Primary strata	Secondary strata
Self-identified Maker With/without formal engineering Education experience (e.g., engineering degree) With/without informal engineering education experience (e.g., robotics team, hacker space) Member of an underrepresented group based on ethnicity or gender	With/without a STEM career With/without a STEM hobby Years of experience as a Maker Age

Makers who have a formal engineering degree were selected to provide insight into how formal engineering education has helped them in their chosen pathway. Makers who have informal engineering education experience (e.g., robotics team) were selected to provide breadth to the study and illuminate how informal education experiences influence engineering pathways and career choices.

At the conclusion of the study, 42 adult Makers had participated with the artifact elicitation interview, and 25 of those participants went on to complete the critical incident interview. The demographic data, as provided by the individual Makers, is shown in Table 4.

**Table 4:** Demographics of interviewees

Age		Ethnicity		Gender	
18–25	4	White	16	Female	17
26–30	10	Asian	11	Male	25
31–40	14	Hispanic	3		
41–60	9	No response	12		
60+	1				
No response	4				

Unlike larger quantitative statistical studies, we seek to identify factors and characterize the landscape through a small number of in-depth examples, but do not claim to generalize or predict more broadly, thereby making a smaller *N* appropriate (Pawley 2013) for the aims of our study. With our qualitative research approach and the exploratory nature of our research questions and theory-building efforts, our number of interviews has produced more than enough “thick description” (Geertz 1994) for the study’s purpose to discern patterns about participants’ pathways through formal and informal engineering experiences.

### Data collection

The study began with administering a screening questionnaire to potential participants to inform the stratified purposeful sampling strategy. Fifteen-minute artifact elicitation interviews (Douglas, Jordan, Lande, & Bumbaco, 2015) based on the method of photo elicitation (Clark-Ibáñez 2004; Harper 2002) were then conducted in person at Maker Faires with each study participant to learn more about the artifacts they were showcasing. A longer, 1-hour, critical incident technique interview (Flanagan 1954) was conducted with each participant in the months following Maker Faire to understand their life pathways.

#### Screening questionnaire

Prior to each Maker Faire, all Makers with information publicly available online were contacted through email and asked to complete a short online screening questionnaire. The questionnaire consisted of short answer questions (see Table 5) and requested contact information and their exhibit location at the Maker Faire. The results were collected in a spreadsheet that was used to select initial participants using the stratified purposeful sampling strategy described above and was also used to contextualize the artifact elicitation and critical incident technique interview questions.

**Table 5:** Screening questionnaire questions

Are you a Maker?	Primary strata
How many years are you a Maker?	Secondary strata
As a Maker, what do you make?	Context for interview
Why are you attracted to Making?	Context for interview
Have you been involved with any group Maker activities?	Primary strata
Have you taken any engineering classes or have an engineering degree?	Primary strata
Do you have an engineering related job/career?	Secondary strata
Ethnicity, gender	Primary strata
Age	Secondary strata

#### Artifact elicitation interviews

Semi-structured artifact elicitation interviews (Douglas, Jordan, Lande, & Bumbaco, 2015), based on the research method of photo elicitation (Clark-Ibáñez 2004; Harper 2002; Morley et al. 2011), were used to elicit “thick description” from participants (Geertz 1973). Interviews were conducted in person with 41 Maker participants and via Skype with one Maker participant to examine the pathways related to engineering that had been followed toward the creation of the artifact on display. The 41 interviews conducted in person took place at each participant’s exhibit booth at the Maker Faire where they were typically interacting with Maker Faire attendees and showing/demonstrating their creation. Following obtaining research consent, approximately 15 minutes was spent with each Maker participant, asking them to describe their artifact, show how their artifact works, describe their process for Making, and describe the knowledge, skills, and attitudes they learned or gained from Making (see Table 6). We asked probing questions about the artifact to elicit “thick description” (Geertz 1973). Questions evolved after each round of data collection based on emergent themes that were discovered during early analysis.

#### Constructivist critical incident technique interviews

Semi-structured constructivist critical incident technique interviews (Flanagan 1954; Klein 1999; Klein et al. 1989) were used to examine the educational and career pathways of Makers and how they intersect with formal engineering education and careers in engineering. Klein used critical incident technique interviews to study decision-making in a variety of fields, and the method has been used very successfully in engineering education research (Adams et al. 2007; Adams et al. 2011; Adams et al. 2010; Adams, Mann, Forin, & Jordan, 2009; Pears et al. 2008; Walther and Radcliffe 2007). This technique aligns well with RQ1 of this study to understand the decision points contributing to pathways intersecting with engineering by providing interviewees with an opportunity to describe the critical incidents in their lives that led them to engage in Making and/or engineering (educational experiences, mentors, deeply held philosophical views, etc.). Following each Maker Faire, all participants who completed an artifact elicitation interview were asked to participate in a critical incident technique interview via email. All Maker participants who were willing to complete the critical incident interview were contacted via Skype. Each interview typically lasted for 1 hour. The interview consisted of questions (see examples, Table 7) designed to examine decision points in their educational pathways and how they relate to engineering. Questions evolved after each round of data collection based on emergent themes that were discovered during early analysis.

**Table 6:** Sample artifact elicitation questions

Can you tell me about what you brought to the Maker Faire? What technology does it use? Can you show me how it works?	Knowledge and skills
What knowledge and skills did you have to learn to make this [insert name of artifact]?	Knowledge, skills
Where did you learn these things?	Lifelong learning
How did you come up with the idea for this [insert name of artifact]? What could you improve in your [insert name of artifact]?	Attitudes

**Table 7:** Sample constructivist critical incident technique interview questions

What would you say “Making” is for you?	Attitudes
Tell me the story of how you became a Maker.	Pathways
How did your educational experience prepare you for the Making you are doing now? Have you found any gaps in your knowledge (e.g., things you wish you would have learned or things you did not learn well enough)?	Lifelong learning/pathways
What is your job? Why did you/did you not pursue an engineering career?	Pathways
Where do you see yourself in 5–10 years?	Pathways

### Data analysis

Throughout the study, part of the research team conducted inductive analysis on the transcribed interviews, which provided feedback to inform questions asked in the interview protocol. Each part of the research team was designated a coding approach so as to reduce bias in the identification of themes.

To examine the pathways of the Maker participants, interview transcriptions were analyzed inductively. Open coding (Corbin and Strauss 2014), theoretical memoing (Glaser 1978), and sorting were used to identify key influences in the participants’ pathways. Sorting and theoretical coding are being used to connect the resultant themes into a theory for the larger work in progress.

#### Data analysis challenges

Characterizing Makers and their pathways is a challenging endeavor. Most Makers do not follow traditional pathways, i.e., education followed by a career in their field; instead, they cross among disciplines, make major career changes, learn diverse skills and knowledge, and so on—often for the purpose of realizing their goals as Makers. The Maker community is built on a culture of acceptance and supporting one another in their individual and group interests and pursuits. As Making goes mainstream, it continues to be a place where people from all backgrounds can gather and showcase their artifacts, whether it be a transformative innovation or an offbeat artifact. The Maker showcase events (including flagship Maker Faires, mini Maker Faires, and other features events) have seen their total attendance grow 24 times since the first Maker Faire; this translates to approximately 22,000 exhibitors and attendees in 2006 to 530,000 exhibitors and attendees in 2014 (Maker Media Inc. 2015). With this growth comes an increase in the diversity of artifacts being showcased and people from different pathways. Browsing by topic on the Maker Faire website will lead you to 70 exhibit topics to explore. These exhibition topics range broadly and include areas such as young Makers, electric vehicles, rockets, art and design, craft, sustainability, and even education (Maker Media Inc. 2014). The Maker Movement is a place for people of all sorts; this type of diversity makes analyzing their life pathways a challenge. Data were analyzed inductively using NVivo in several iterations. Specifically, researchers explored the Maker’s life pathways for critical incidents, including major milestones and key influences, and inductively coded them.

## Results

### A view from the top

**Fig. 2:**
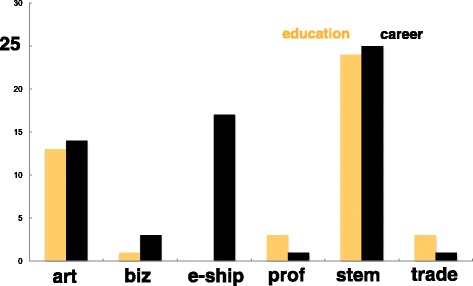
Educational backgrounds and careers of the Maker participants (consolidated categories)

The inductive analysis revealed the broad range of Makers’ reported educational backgrounds and careers. Makers were educated in a range of fields, including fine art, trade school, engineering from bachelors to PhD, game design, and creative writing. Their career experiences were similarly varied and included professionals, academics, civil servants, and tradespeople. Makers often move between multiple careers through the course of their lives. Of the 42 participants, including those who reported having more than one career, 25 have formal engineering education experience, 13 have an engineering degree or are currently studying toward an engineering degree, and 21 have informal engineering education experience (e.g., robotics clubs). Of the same participant pool, 22 reported having an engineering-related career and 7 reported *not* having an engineering-related career. Participants often reported multiple careers. A given participant might have been a machinist, engineer, and artist at different times of their lives, for example. Consolidating the responses for educational backgrounds and careers into like categories reveals that the frequency of educational background and career for the art and STEM categories is somewhat proportional. Additionally, most Makers have STEM and/or art education backgrounds and careers, in addition to being involved with entrepreneurship in their careers. From this vantage point, it seems as though the Makers’ pathways are mostly linear (e.g., an individual who has an educational background in STEM has a career in STEM). Looking across the educational background to a career for the individual participants reveals that 34 pathways are linear, with 16 involving entrepreneurship in the career. Two pathways show crossover between art and STEM, and four pathways show crossover between multiple categories (e.g., tradesman, professional, business). Twenty-four of the 42 interviewees had careers or education experiences bridging across multiple categories. A summary of educational backgrounds and career pathways is shown in Fig. 2.

As shown in Fig. 2, the majority of Makers we interviewed had some experience, educational or career, in art or STEM. However, a broad overview like the one presented above fails to capture the nuance of how any given Maker made their way from education to career, from career to their second career, or their journey of turning a hobby into an entrepreneurial venture. To gain deeper insight into how individuals might make their way into Making and how they might pass through engineering, more specific analysis was required as is explored below.

### A new direction for analysis: parallel pathways

Through the inductive analysis, a critical theme emerged where Makers from different educational backgrounds and with different careers (e.g., art, STEM, business) were making artifacts that had a similar purpose (see Table 8). These groupings of Makers, based upon the artifacts they made, became an interesting technique to study the complexity of Makers’ pathways. Two of the broader case areas are listed below along with the education and career backgrounds of the individual makers. Within these areas, we can compare makers with wildly different relationships to engineering who produce similar artifacts. This process for comparison is intended to prompt new questions, uncover new dimensions, and produce alternatives (Khan and VanWynsberghe 2008). This is important when studying pathways to consider the multiplicity of ways that Makers arrive at Making and how it intersects with engineering. With this new direction, artifacts produced by the Makers were grouped based upon similarity. Two examples of artifact types, and their more specific sub-categories, are shown below (see Table 8). The two specific categories we explore further in this paper are bolded, large-scale toys, and music (electronic). The focus for analysis within each case was on the events, activities, and processes that were key to the Makers’ pathways (Khan and VanWynsberghe 2008). While the data from screening questionnaires provides an interesting view at the major milestones along the Makers’ life pathways, it tells us little about the specific events that occurred in their pathway and the skills that they gained through the activities and events (Table 8).

**Table 8:** A list of cases and the Makers that comprise them

Case	Educational background	Career
Toys		
Electronic		
E-textiles	BS, ME, and PhD in Mechanical Engineering	Research Engineer
Games	Fine art	Masters student in Game Research
Science toys/jewelry	PhD in Chemical Biology	Scientist
Machine art/kinetics	Electrical Engineering	Retired
Building/circuits	BS, MS in Electrical Engineering	Entrepreneur
Interactive		
Interactive coding	New media	Advertising, engineering freelance
Augmented 3D games	Game design	Entrepreneur
Large-scale		
Articulated mannequins	Mechanical Engineering	Mechanical Engineering
Carnival	Fine art	Masters student in Art
Athletic activity	Game design	Game designer
Physical		
Puppets	Pilot, police officer, apprentice	Entrepreneur
Paper fractal activity	Science	Masters student in Science
Music		
Speakers	Manufacturing Engineering	Entrepreneur
Noise band	Art, sculpture—BFA	Art professor
Robotic symphony	ME and Electronics	IT director + entrepreneur
Non-electronic		
Wooden bicycles	Woodworking	Small business
Origami kayaks	Engineering, architecture	Entrepreneur

Two cases of parallel pathways, (1) musical artifacts and (2) large-scale interactive artifacts, are presented in this paper to demonstrate the multiple, parallel pathways that Makers take to making their artifacts and the contextual events and activities that are critical to the direction of these pathways. These cases were selected as illustrative examples for this paper because the Makers within each case have different educational backgrounds and different careers, despite making artifacts of similar engineering sophistication. Examining the similarities and differences in the pathways within each case illustrates how Making intersects with engineering and how engineering pathways might be broadened. The findings in this section are highly contextual, dependent upon thick descriptions, and have not undergone comparative analysis across the cases. In-depth comparison within the cases and across the cases needs to be performed to better understand the pathways of Makers and how they intersect with engineering. However, the cases provide a unique starting point for discussion of the pathways of Makers.

### Case 1: musical artifacts

**Fig. 3:**
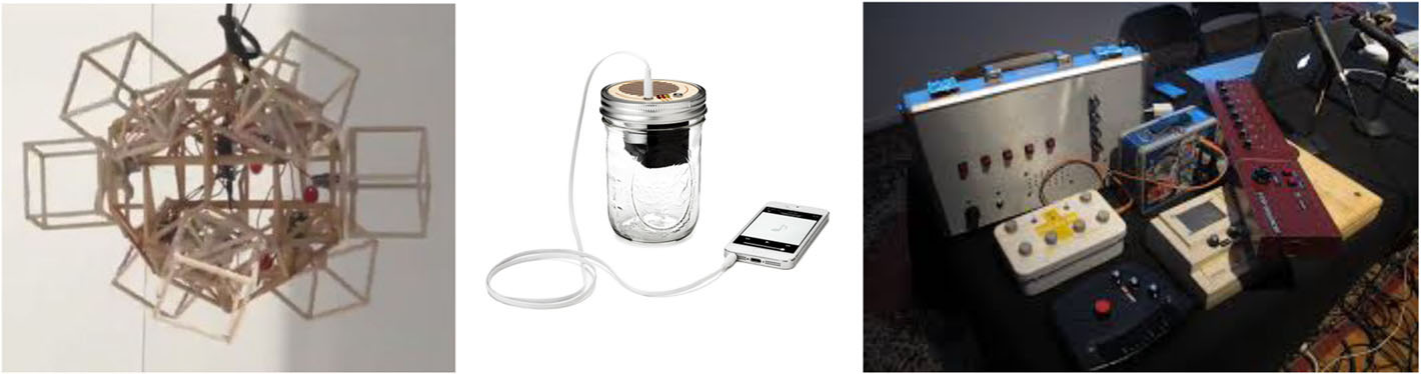
Musical artifacts of the Makers (Alejandro, Cane, and Stephen, respectively)

**Fig. 4:**
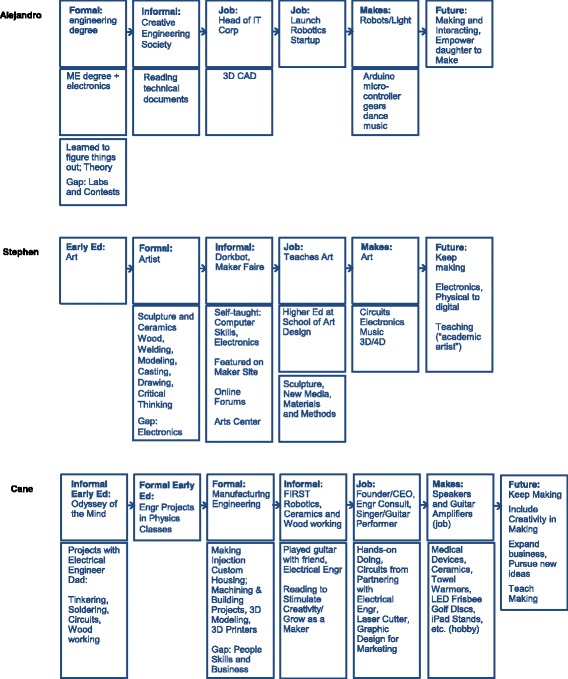
Parallel pathways for the Makers of musical artifacts

Alejandro, Cane, and Stephen (*pseudonyms*) make musical artifacts (see Fig. 3). Alejandro makes robots that dance to micro symphonies. Cane makes musical speakers from up-cycled products (e.g., soda cans and lunch boxes) for his growing business. Stephen makes musical instruments from discarded products (e.g., children’s toys) for improvisational performances with an organized group. Each of these Makers used technical knowledge and skills to bring their artifacts to fruition; however, each of their pathways to technical activities is different. Through three stages of data collection (screening questionnaire, artifact elicitation interview, and critical incident interview), new dimensions of their individual pathways were uncovered (see Fig. 4).

Alejandro’s pathway seems somewhat standard for engineering; he had 6 years of engineering schooling, which allowed him to obtain a Mechanical Engineering degree with an emphasis in Mechatronics. He attributes his theory-heavy formal engineering education experiences to enable him to “look for things and figure out things.” However, he wished his formal engineering education had more lab experiences and contests because “it is also frustration that teaches you a lot”. Like many engaged engineering students, he sought out informal engineering education experiences by participating in an engineering society; specifically for Alejandro, he would build and present Rube Goldberg devices at science fairs. Alejandro became familiar with the label “Maker” when he was invited to present his robots at the Maker Faire. His drive for Making came from the ability to go “against the socially accepted project in engineering school.” He pursued the intersection between art and engineering through his Making, allowing dance and music to inspire the creation of his robots. When his peers in engineering school tried to label him as an artist for making non-traditional engineering devices, he would respond to them saying, “I’m not an artist. I’m just an engineer that creates moving things.” When asked what his future aspirations are, Alejandro reminds us that his artifact at the Maker Faire was the first performance for his dancing robots and that he has four more performances to make to represent different forms of dance (e.g., Bolero). While working a technical day job, he has launched a robotics start-up to pursue his interests in dancing and musical robots. Alejandro’s pathway reflects one of a traditional engineer inspired and informed by his art.

Cane’s pathway is another example of one that seems standard for engineering; he has an engineering degree in manufacturing engineering and is working as a consultant using the knowledge he gained from his engineering education while also running a business that relies upon manufacturing and electrical engineering knowledge and skills to make musical artifacts (e.g., modeling, casting, using solid works). Like Alejandro, Cane participated in informal engineering education experiences; however, Cane’s focus was on his interest in teaching, so he sought out experiences in this vein (e.g., being a mentor for FIRST Robotics). His passion for the arts also led him to experiences outside of engineering (e.g., teaching ceramics and woodworking). A defining life event for Cane was growing up with a dad who was an electrical engineer and working on projects with him while learning technical and non-technical skills (e.g., soldering and woodworking). He also attributed engineering projects in the science classroom as shaping his pursuit of an engineering degree. Cane’s identity as an “inventor” drove him to manufacturing engineering whereby he learned “how to make things.” Once again, his passion for the arts led him to launch a business where he could combine his engineering knowledge and skills with music. Unlike Alejandro, he did not see a disconnect with engineering and the work that he is doing; rather, he wishes that his formal engineering education could have been extended to include developing interpersonal skills and business skills to enable people to leverage their ideas and pursue their goals. According to Cane, his future will include continuing to make the things he is making, to expand his business to other products, and to get involved with teaching again. Cane’s pathway reflects one that was driven by early childhood experiences and a pursuit to use his engineering education to implement his art.

Stephen’s pathway is dissimilar to that of Cane and Alejandro in that he is trained as an artist and works as a professor of art at a school of art design. Stephen points to positive experiences with art from an early age and how art “engaged” him. To Stephen, interacting with the world around him and learning new skills and knowledge is critical. He pursued the arts because “it is an excuse to learn everything.” His knowledge and skills span a wide array (e.g., woodworking, modeling, casting, materials, and drawing). Through his formal education in sculpture and ceramics, he became interested in technology. “I’ve always been interested in technology and oddly I got into sound as an art form from sculpture.” Through this interest, he began to learn about electronics and made “boxes that make noise.” He became involved with informal education activities that support technical activities, including a group dedicated to doing things with electronics. From his musical artifacts, he formed a group that mimics that of jazz improvisation, where the members “have a relationship with each other even before they get on stage and have a relationship with the instruments.” When asked how he learned to make his musical artifacts, he points to his formal art education and self-directed learning. He points out that he is interested in engineering and that he would have pursued it had it been available to him. “If I had had actually somebody advising me when I was getting out of high school and going to college and I knew engineering as a career, I might have been an engineer to be honest, because I love engineering as a concept of being aware of your world and being in your world.” To Cane, engineering is “taking that knowledge of the world and creating because it is a very creative endeavor, creating something new that exists in that world that changes how people interface with that world.” Recognizing the similarity with art, he says, “that’s what art is all about at a certain level.” His future aspirations are to keep Making, specifically electronics, and to continue being an “academic artist” that enables him to pursue Making with technology. Although his pathway differs from that of an engineering pathway, he has overlap in the knowledge and skills he has obtained and his purpose for Making.

### Case 2: Large-scale interactive artifacts

**Fig. 5:**
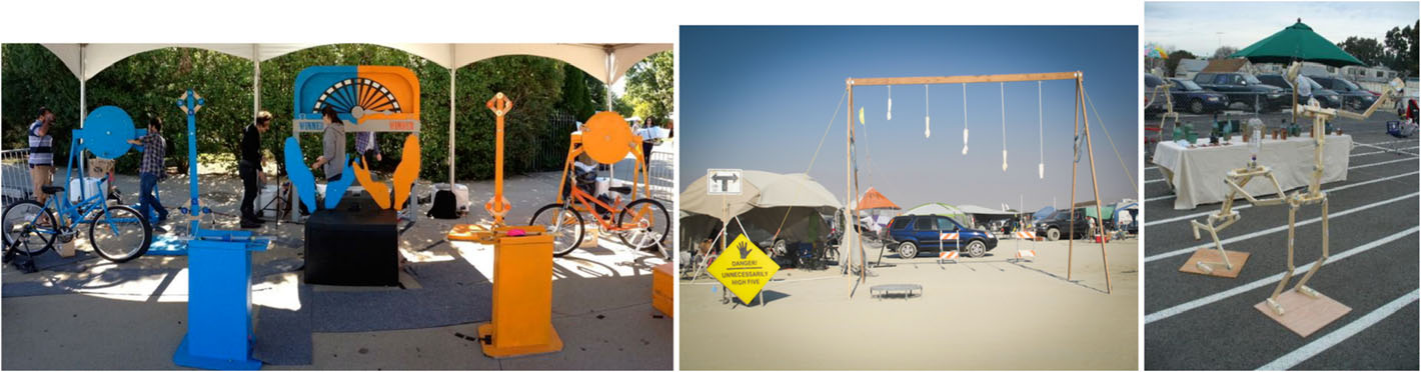
Large-scale interactive artifacts of the Makers (Heather, Jack, and Mark, respectively)

**Fig. 6:**
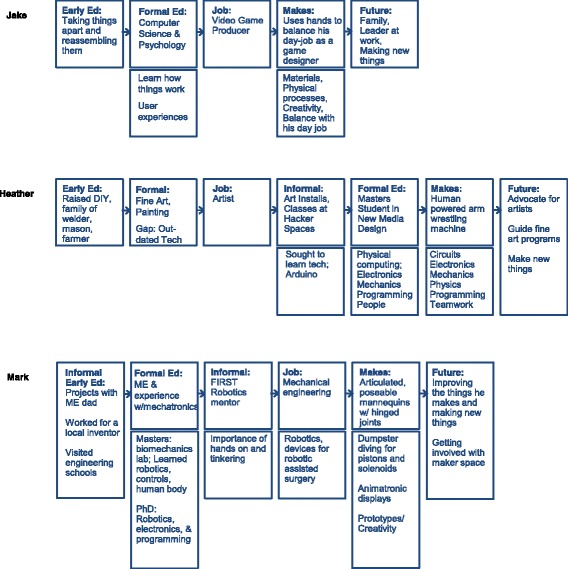
Parallel pathways for the Makers of large-scale interactive artifacts

Heather, Jack, and Mark (*pseudonyms*) made large-scale interactive artifacts to exhibit at the Maker Faire (see Fig. 5). Heather made a human-powered arm wrestling activity, whereby two mechanical arms are powered by human energy (e.g., bicycles and turn cranks). Jack made a large-scale, high-five activity where Maker Faire attendees can experiment with how high they can jump. Mark made articulated, life-size, stick-figure mannequins with hinged joints that Maker Faire attendees can position to create poses and stories. Each of the Makers collaborated with like-minded individuals to bring the artifact to fruition. Heather worked with fellow graduate students, while Jack and Mark worked with friends of the same discipline. All three of these Makers’ artifacts were created in the spirit of fun and to showcase at the Maker Faire; the artifacts were outside of the Makers’ normal realm of Making. Like in the first case, each of these Makers used technical knowledge and skills to bring their artifacts to fruition; however, each of their pathways to technical activities is different. Through three stages of data collection (screening questionnaire, artifact elicitation interview, and critical incident interview), new dimensions of their individual pathways were uncovered (see Fig. 6).

Heather’s pathway is intertwined with art and technical activity. Heather grew up in a do-it-yourself environment, where all members of her family made things with their hands (e.g., her father was a welder, and her mother made the family’s clothes and grew their food). She encountered difficulties in her life after graduating high school, but art rescued her. She entered into an undergraduate program for fine art and became trained as a painter. She pursued her painting career for 10 years. As she worked in art galleries, she became dissatisfied with painting because to her, it seemed “outmoded and, technologically speaking, it had died a long time ago.” She was drawn to interactive work because of its “engaging and thought-provoking” characteristics. This prompted a path of self-directed learning to adopt new skills in technology; she took courses at local hacker spaces to learn Arduino, robotics, and kinetics. During this shift in her pathway, she never forgot the role that art has in her life. “I owed my life to my art because it got me out of situations that my peers are still embroiled in. No matter what I do, I always owe my art my best.” With this dedication to art, she began to plan how she could appropriate her developing skills with Arduino, robotics, and kinetics to be “a better artist.” During her self-directed learning, she found the local programs lacking for an individual like herself who had no technical foundation. She entered into a graduate program at an interactive art school. During this formal education experience, for which she was still enrolled in during the time of the Maker Faire, she acquired the skills and knowledge needed to know to propel her forward (e.g., mechanics, electronics, fabrication, and production). Preparing to graduate, she recognized “so now I know what to ask and how to ask.” She gives credit to her graduate program for “getting her over that bump” and exposing her to like-minded individuals who can participate and contribute in her Making. To Heather, Making is “a verb; it is finishing something.” She sees the Maker Movement as a place that brings together different sects, from “those that just want to build” to those that want to engineer and “make sure all the technical components are there.” She sees herself as the jock. When asked about her future aspirations, she says she would like to (1) “be an advocate for artists with art knowledge and purpose and intent”, (2) “guide more traditional institutions of fine art into this idea to give artists digital tools,” and (3) “for my own selfish intent, I want to make things that are awesome, things that I see in my head and make them happen.”

Jack’s pathway represents a blend of technical training, Making, and design. He has a degree in computer science and psychology and works as a video game producer. He focuses his responses to the interview questions on design, creativity, and implementing ideas. He credits his dual degree with enabling him to know “how things work programmatically and how people might interact with the [artifacts and games].” When asked about the specific knowledge and skills he learned from his formal education, he cannot recollect specific examples and discloses that the field of computer science has changed so much since he went through his degree program. He identifies as always being a Maker and references early childhood experiences with being “creative” and taking “whatever is in my head and make into something real.” As a child, he would take things apart and put them together again. He uses Making as a creative outlet to work with physical processes and to use his hands; this balances his computer-heavy work with game design. “When I’m building it, I’m working with a lot, you know, I’m working with tools and actually like getting calluses on my hands as opposed to when I’m Making video games it’s all virtual.” Through Making interactive artifacts, he learns about materials, like conduit, and processes. He purposefully selects projects for Making that are less time intensive to allow for time for his young son and work, but relies upon Making to “push creativity in a different direction.” He also tries to keep his Making separate from his job as a game producer, but acknowledges that they inform one another, specifically with “creativity and having to create access to tools.” He identifies himself as “more of a designer and a visionary and about user experience.” Recognizing how Making has shifted his mindset, he now considers himself an artist. Before Making became mainstream and he became familiar with the label, he considered himself “as a creative, but not necessarily an artist.” He now feels like he is an artist because he has a “skill set” and is making ideas “into real things.” In the future, Jack hopes to be a leader at work, raise his family, and continue to make new things. His views of art and technical work influence his directions in life; this is interesting to compare to Heather, whose pathway has also been influenced by her views of art and technical work.

Of all the pathways presented, Mark’s pathway might seem the most traditional for engineering; he has mechanical engineering degrees (BS, MS, and PhD) with experiences in Mechatronics and is a practicing mechanical engineer with a career in robotics. His father was also a mechanical engineer and liked to do hands-on projects together, such as radio-controlled airplanes, submarines, solar-powered mobiles, and building things from Erector sets. From an early age, he had foundational experiences with engineering, like working for a local inventor who “had invented a machine that watered automatically trays of alfalfa sprouts.” When it was time to select a university, his dad flew with him to reputable engineering universities for tours. During graduate school, he worked in a biomechanics lab where he was “introduced to human anatomy” and learned how the joints and muscles work. During his Master of Science program in engineering, he extended his knowledge of robotics and controls, and during his PhD program in engineering, he studied electronics, robotics, and programming. He uses his knowledge and skills for his job, designing devices for robotic-assisted surgeries. He credits the blend of his knowledge and his interest in “combining engineering and art” for the reasons he makes the articulated mannequins and animatronics features. The artifact initially began as a hobby: “I have had a career in robotics, and I’ve had kids, and I enjoy entertaining kids particularly at Halloween time.” However, “sharing it with other people” drove him and his friend to bring the artifacts to the Maker Faire. He has been showcasing these artifacts for 8 years, each time making them better. “People loved them; they tore them apart; they broke all the wires in them, so we figured we would make them better. Each year we have improved the mechanical design of them.” In addition to his recreational Making and his engineering career work, he mentors high school students to promote “hands-on learning through robotics.” When asked about gaps in his education experiences with engineering, he references a list of technical topics that he wished he had more exposure to, including statistics, programming language, and digital electrons. When asked if there are any other gaps, he refers to his “artistic talent” and that he never pursued art courses, but learned it on his own. In the future, Mark aspires to continue to improve the things he makes, make new things, and teach at local tech shops. His pathway is familiar to engineering. It is starkly different than that of Heather’s and overlaps with Jack’s in his dedication to make physical artifacts.

### Summary

The inductive analysis of data revealed a theme whereby individuals of differing educational backgrounds and careers were making similar artifacts. Upon exploration of Makers’ pathways, as grouped by the types of artifact made, revealed an intertwining of experiences that spanned across engineering and art as well as traditional and extracurricular experiences. Makers were willing to go outside of the realm of traditional engineering to fuel their abilities to make. The cases of parallel pathway cases provide lessons for how to explore the rich, nuanced pathways of Makers, and how they span across multiples disciplines, experiences, and activities.

## Discussion

The inductive analysis of data provided a rich narrative of each Maker’s pathway. With each set of data collection, there was something to be learned about the Makers’ pathways. This was useful in understanding the breadth of Makers’ pathways and how they overlap with one another and intersect with engineering. Overall, Making can offer valuable insight into how to identify practices that promote the access and success of a larger and more diverse population of students within engineering. Opportunities may exist to export interview techniques for other uses. Artifact elicitation interviews, for example could be used with entrepreneurs to better understand to the values and skills behind product development or with robotics engineers to gain insight into the creation of a robot. Combining this with critical incident interviews could offer new insights that relate to new opportunities for pathways into engineering. The life pathways of Makers can begin to change the conversation to highlight the efficacy and the possibility for those who are engaged in Making and seeking meaning and impact through their studies.

Specifically, from the methods of this study, we get a snapshot, though a very detailed snapshot, of one artifact at one point in time. The critical incident interview is in relation to this one artifact and does not provide a fully fleshed out life history. However, when aggregated, these snapshots form a broader picture, which describes the community of Makers in a useful way and allows us to see possibilities for broadening engineering pathways.

The screening questionnaire was useful in unearthing the major milestones along the individual’s pathway (e.g., degrees, clubs, and what they make). However, the responses from the screening questionnaire provided no context for Making. With this dataset alone, individual pathways might have looked linear (e.g., an engineering major working in a technical field) and overlooked other important events and turning points in the pathways.

The artifact elicitation interview provided a context for the Makers’ pathways. By providing an opportunity to conduct a live-action interview (i.e., interviewing each Maker with their artifact), we were able to ask probing questions about the artifact and the knowledge, skills, and attitudes that the Maker learned from Making. By watching the Maker operate and showcase their artifact, we gained an understanding of the functionality of the device and technical and non-technical components, which informed probing interview questions. From the probing interview questions, Makers had the opportunity to bring up experiences they have had in their pathways (e.g., examples of learning knowledge and skills through self-directed learning and informal education activities). The setting for artifact elicitation interviews was exciting. The Maker Faires were filled with people that had enthusiasm for the artifacts. Makers were ready to show their artifact and interact with the public. These interviews could only last 15 min in order to be respectful of the participants’ time and allow them to interact with other attendees.

The critical incident interview further extended our understanding of the Makers’ pathways by providing an opportunity to ask the Makers to walk us through their critical points in their life (e.g., education, career, and future aspirations). By conducting this interview via Skype, the Makers were able to give more of their time to respond to questions and were able to elaborate upon their pathways to Making.

Identifying appropriate methods for studying pathways remains a challenge. In the case of the Makers, examining the pathways is a nuance, non-linear, and includes pivots due to defining life events (e.g., engineer to entrepreneur). Utilizing a qualitative research approach of constructivist grounded theory, using artifact elicitation interview and critical incident interview methods, is a useful contribution to telling the story of the life path and providing a deeper look at the knowledge and skills that are learned that may be related or useful to engineering.

## Conclusions

Adult Makers show us that anyone—credentialed or simply curious—can participate in engineering as a hobby and/or a profession and that engineering is often not a binary category where one is performing either as an engineer or a non-engineer. Instead, we see a wide range of actors with widely varying skill sets engaged in engineering activities. More importantly, we see non-engineers valuing engineering expertise and knowledge in new ways through the lens of Making. Similarly, engineers are either discovering new outlets for their existing engineering skills or learning new engineering and non-engineering skills in pursuit of their passions. While this work is still ongoing, it may showcase some ways in which engineering education can be enhanced to better reach the goals outlined in *The Engineer of 2020*. If adults are finding interdisciplinary projects framed by personal interest as a way of learning practical ingenuity, creativity, and some analytic skills, perhaps there is a way educators can harness student passions (interests and motivations?) in a similar manner to achieve similar results. Furthermore, the interest shown by non-engineers for learning engineering as adults, along with existing engineers expanding their scope of knowledge, could have ramifications in the adult education sector. Perhaps, there is a currently unmet demand for adult education in engineering, which could be met by existing universities offering, for example, night courses on circuits for non-engineers. And, as demonstrated by the quote:

If I had had somebody actually advising me when I was getting out of high school and going to college and I knew engineering as a career, I might have been an engineer to be honest, because I love engineering as a concept of being aware of your world and being in your world.

Engineering is perhaps being presented to young adults in a way that obfuscates the creativity and impact that engineering can have in the world around them. When adult artists suggest they would have chosen different career paths if they had been presented with engineering as a creative career which embraces practical ingenuity, ethics, and communication as well analytic skills, then there is perhaps a better way going forward to market engineering to incoming students.

Through an in-depth exploration with qualitative inquiry, a new perspective is offered that can inform us of how access to engineering from qualified learners may be improved. Makers are self-directed learners and have diverse technical and non-technical backgrounds; many may be qualified to enter engineering majors. The study of Makers unveils opportunities and new dimensions for access and migration to engineering.

A more inclusive vision of engineering crossed with Making could build future engineering capacity as well as raise awareness to the general public of the work and impact such work offers. If we return to the Figueiredo (2008) four-dimensional transdisciplinary model of engineering, Makers interviewed showed strong capabilities in “design knowledge” and “knowledge from doing” categories. This combines with a lower demonstration, but high interest, in the “basic science knowledge” and “social science knowledge” categories. Makers are developing strong abilities in knowledge domains that relate to engineering as well as interest in basic science knowledge and social science knowledge as one might find in an ideal engineering student. Within this context, the activities of and people engaged in Making may make for engaged learners with a broad level of expertise across the four-dimensional transdisciplinary model. It suggests that interest in Making, and in particular, in knowledge of design and knowledge from doing, can lead to greater interest in learning the basic science as applied to engineering. This alternative entry and pathways into being interested in engineering as a way of Making may attract a broader base in a potential audience by way of age, prior education, and interest that the prototypical current engineering student.

The pathways presented in this study are far different than what many early engineers imagine. Instead of a linear progression of high school to college to work to professional engineer qualification to retiring someday, these pathways show that engineers can be much more broadly interdisciplinary and engage with multiple fields, both within engineering and with disciplines such as art or business. Engineering is often perceived as an activity lacking in creativity and, for some, meaning. However, interdisciplinary interactions with engineering can show adults, and presumably youths, that engineering is a creative way to interact with and affect the world around you and could be a way to improve access to engineering. These pathways show that engineering may be approachable for non-engineers involved in Making and technical activities. The examples of Maker pathways could be used as example stories for how students may pursue engineering in the future.

The stories and life pathways of adult learners engaged in Making can offer valuable insight into how we might identify practices that promote the access and success of a larger and more diverse population of students. We do not equate engineering students, practicing engineers, and Makers completely but find the possible overlaps and stories of pathways within to be possible for a transformational change in our field. Makers are engaged in activities that embody the Engineer of 2020 (e.g., lifelong learning, creativity, and practical ingenuity). By studying Makers, we can consider the multiplicity of pathways into engineering majors and careers.
